# Cytokine Storm in COVID-19: “When You Come Out of the Storm, You Won’t Be the Same Person Who Walked in”

**DOI:** 10.3389/fimmu.2020.02132

**Published:** 2020-09-02

**Authors:** Vanessa Castelli, Annamaria Cimini, Claudio Ferri

**Affiliations:** Department of Life, Health and Environmental Sciences, University of L’Aquila, L’Aquila, Italy

**Keywords:** cytokines, SARS-CoV2, inflammation, coronavirus, acute respiratory distress, severity

## Abstract

In December 2019, a novel coronavirus, COVID-19, was discovered to be the causal agent of a severe respiratory infection named SARS-CoV-2, and it has since been recognized worldwide as a pandemic. There are still numerous doubts concerning its pathogenesis and particularly the underlying causes of the various clinical courses, ranging from severe manifestations to asymptomatic forms, including acute respiratory distress syndrome. The major factor responsible for acute respiratory distress syndrome is the so-called “cytokine storm,” which is an aberrant response from the host immune system that induces an exaggerated release of proinflammatory cytokines/chemokines. In this review, we will discuss the role of cytokine storm in COVID-19 and potential treatments with which counteract this aberrant response, which may be valuable in the clinical translation.

## Introduction

For the third time, a zoonotic coronavirus has crossed species boundaries to infect humans. Initially, the virus was detected in people exposed to seafood. First reports revealed that human-to-human transmission was impossible or restricted; it is now clear that such transmission occurs, though the underlying mechanisms are still unclear (1). In December 2019, this coronavirus was detected for the first time in the respiratory tract of patients with pneumonia in Wuhan, Hubei, China, and it was identified as a new β-coronavirus (nCoV). This novel coronavirus was subsequently named as Coronavirus-2 disease (COVID-19), and it leads to Severe Acute Respiratory Syndrome Coronavirus 2 (SARS-CoV-2). COVID-19 has afflicted about 5 million people worldwide, and it was recognized as a pandemic by the World Health Organization (WHO) in March 2020 (2, 3).

The effect of COVID-19 can encompass anything from asymptomatic disease to critical infection, with 661,244 deaths reported worldwide to date (July 31, 2020) (4) and more than 30% of hospitalized patients needing mechanical ventilation in intensive care units (5, 6). Death rates depends on aging and presence of comorbidities (including obesity, diabetes, cardiovascular problems, cancer and hypertension) (5, 7). Patients with severe COVID-19 showed multi-organ failure and rapid advancement of lung infiltrates, which is concomitant with a sustained release of inflammatory cytokines and biochemical makers of inflammation. The cytokine storm could be on the basis of the difference between asymptomatic and patient with severe symptoms (6, 8–11). So far, seven human coronaviruses have been found, comprising α-types (HCoV-229E and HCoV-NL63), β-types [SARS-CoV, Middle East respiratory syndrome coronavirus (MERS-CoV), HCoV-HKU1, and HCoV-OC43] and the present 2019-nCoV epidemic. Regarding their pathogenicity, human coronaviruses are divided into being moderately and severely pathogenic, and these include SARS-CoV (12), MERS-CoV (13), and SARS-CoV-2 (14). The entire human population lacks immunity to SARS-CoV-2 and is thus susceptible to the novel coronavirus. To date, no exhaustive studies have been reported about the immune host response to SARS-CoV-2, and we consequently need to relate to previous findings on other CoVs.

## Aberrant Host Immune Response

The underlying mechanisms of severe infection in patients affected by SARS-CoV-2 are still unclear, and the progress of the severe form does not seem to be exclusively related to a viral titer and may include defective interferon responses (15). An excessive inflammatory response to SARS-CoV-2 represents the main cause of disease severity and death in COVID-19 patients (16), and it is characterized by acute lymphopenia, elevated levels of circulating cytokines, and substantial mononuclear cell infiltration in the lungs, spleen, kidneys, lymph nodes (6), and heart (17), as revealed in post-mortem exams.

It is well-known that cytokines perform a key function in the immunopathology during viral infection. The first response against viral infection is a synchronized and fast innate immune reaction. Extreme and uncontrolled immune reactions may, however, trigger immune impairment in the human body (18–20). In patients affected by SARS-CoV-2, the proinflammatory response and, in particular, the cytokine storm represent a centerpiece of COVID-19 pathogenesis, causing great destructive consequences for the host. When the immune system is not more able to counteract the virus and to conclude the inflammatory response, the aberrant production of the cytokines led to macrophage hyperactivity, with consequences for the whole body, including fever, anemia, and organs malfunction. At some point, the cytokine storm becomes unstoppable, leading to irreversible end-organ dysfunction and even death (21, 22).

*In vitro* studies reported that, at the initial phase of SARS-CoV infection, a delayed release of chemokines and cytokines appeared in macrophages, airway epithelial cells, and dendritic cells. In the following phases, cells secrete elevated quantities of proinflammatory cytokines (including interleukins and tumor necrosis factor) and chemokines [C-C motif chemokine ligand (CCL)2, 3 and 5], which is in parallel with low quantities of antiviral factors interferons (INFs) (23–26).

Like SARS, MERS coronavirus infects human respiratory epithelial cells, dendritic cells, and peripheral blood monocyte-derived macrophages, inducing delayed but elevated quantities of chemokines and proinflammatory cytokines (25, 27). In the following stages of the infection, plasmacytoid dendritic cells, but not dendritic cells and mononuclear macrophages (28), are induced to produce a large amount of IFNs. Indeed, serum chemokine and cytokine levels are considerably more elevated in patients with severe MERS than patients with moderate MERS (29), associated with higher number of monocytes and neutrophils in lung tissues and blood of these patients; these cells may thus be involved in the pathogenesis (26, 30). Comparable events have been reported in patients with SARS-CoV infection (26, 31–33).

The delayed release of IFNs during the infection impedes immune system activation against the virus (18). Subsequently, the rapid increase in cytokine and chemokine release stimulates different inflammatory cells, including monocytes and neutrophils, causing an excessive infiltration of the inflammatory cells into lung tissues with consequent lung damage. An over-response of the infected cells seems to be at the basis of MERS or SARS pathogenesis.

Animal models help dissect the role of chemokines and cytokines in the immunopathology after coronavirus infection. Notably, SARS-CoV-infected old non-human primates showed higher probability of developing an excessive inflammatory response compared to young primates characterized by more severe pathology (34). The immune overreaction rather than virus titer is crucial in determining the old non-human primates death (34). Comparably, BALB/c mice infected with SARS-CoV showed higher severity in old mice, which is associated with early and strong upregulation of the acute respiratory distress (ARDS)-related inflammatory gene signals (35). The fast replication of SARS-CoV in these animals leads to the delayed release of IFNs in parallel with the invasion of various mononuclear macrophages (18). These macrophages receive activating signals through the IFN-α/β receptors on their surface and release monocyte chemoattractants (such as CCL2, CCL5, and CCL7), resulting in the additional accumulation of mononuclear macrophages. Furthermore, mononuclear macrophages stimulate higher release of proinflammatory cytokines [ILs and Tumor Necrosis factor (TNF)], thus increasing the severity of the disease. Indeed, it has been demonstrated that neutralizing TNF or reducing the inflammatory macrophages in mice protected from SARS-CoV infection, and INFs or macrophages led to T-cell apoptosis, ulteriorly preventing viral infection (18). In the light of this, it has been postulated that COVID-19 showed similar behavior to other CoVs.

## Acute Respiratory Distress

Since the first reports on COVID-19 disease, it appeared clear that ARDS has led to a relevant number of deaths among infected patients. ARDS should be considered an immune-mediated clinical consequence in SARS-CoV-2, similarly to what described for SARS and MERS infections (17).

The “cytokine storm” concept is derived from the observation that COVID-19 patients requiring intensive care unit admission presented elevated circulating concentrations of CXCL10, CCL2, and TNFα as compared to those in which the infection was mild or moderate (36, 37).

Furthermore, elevated levels of IL-1, IFN-γ, IP-10, and monocyte chemoattractant protein 1 (MCP-1) have been detected in patients with COVID-19. These inflammatory cytokines may stimulate the T-helper type 1 (Th1) cell activation (38). The Th1 response is a crucial event in the immune system response (39). In contrast to SARS patients, however, individuals affected by COVID-19 also showed higher levels of Th2 cell-secreted cytokines (i.e., IL-4 and IL-10), which inhibit the inflammatory response. Serum levels of these cytokines in COVID-19 patients are related to higher severity the disease (26). In addition, COVID-19 patients in intensive care units showed elevated serum levels of granulocyte colony-stimulating factor, IP-10, TNF-α, MCP-1, and macrophage inflammatory protein 1A respective to patients from general areas (38). The cytokine storm occurred in response to SARS-CoV-2 infection and induced the upregulation of Natural killer group 2 on Natural Killer and cytotoxic T cells. This increase inhibited the function of these cells and counteracted cytokine release (40–42).

Another effect of the fast-viral replication and of the strong proinflammatory response is the induction of apoptosis in pulmonary endothelial and epithelial cells. In particular, IFNs cause inflammatory cell infiltration through mechanisms, including TRAIL (TNF-related apoptosis-inducing ligand)–death receptor 5 and Fas–Fas ligand (43–45).

Lung endothelial and epithelial cell apoptosis damages the respiratory microvascular and alveolar epithelial cell barriers, leading to alveolar edema, vascular leakage, and, finally, causing hypoxia in the entire body. Consequently, inflammatory mediators are at the basis of the pathogenesis of ARDS. ARDS is the primary cause of mortality in patients affected by SARS-CoV or MERS-CoV (46, 47). It is known that several proinflammatory cytokines (IL-6, IL-8, IL-1β, and granulocyte-macrophage colony-stimulating factor), chemokines [such as CCL2, CCL5, IFNγ-induced protein 10 (IP-10), and CCL3], and reactive oxygen species all participate in the development of ARDS (48–50).

After SARS-CoV infection, a high virus load and exaggerated immune response lead to an inflammatory cytokine storm, accompanied by immunopathological alterations in the lungs and then in other organs. ARDS and multi-organ malfunction appeared quickly, leading to death within a short period (51). Overall, the cytokine storm is considered to be one of the main causes of ARDS and multi-organ failure (52).

ARDS pathogenesis implicates inflammatory damage to the alveolus–capillary membrane, with consequent improved pulmonary permeability and elevated exudation of protein-rich fluid into the airspaces, inducing respiratory insufficiency. Current management of COVID-19 is supportive, and respiratory failure from ARDS is the leading cause of death (16, 53). Secondary hemophagocytic lymphohistiocytosis (sHLH) is an under-recognized hyperinflammatory syndrome characterized by a fulminant and fatal hypercytokinemia with multiple-organ failure. In adults, sHLH is most commonly triggered by viral infections (54) and occurs in about 4% of sepsis cases (55). Key characteristics of sHLH, comprising chronic fever, hyperferritinemia, cytopenia, and pulmonary involvement (including ARDS), appeared in approximately 50% of patients (56). A cytokine profile resembling sHLH is associated with COVID-19 disease severity, characterized by increased IL-2, IL-7, granulocyte-colony stimulating factor, INF-γ inducible protein 10, CCL1, macrophage inflammatory protein 1-α, and TNF-α (38).

A recent retrospective, multicenter study of 150 confirmed COVID-19 Chinese patients revealed that predictors of mortality involved higher IL-6 and ferritin levels (mean 1,297.6 ng/ml in non-survivors vs 614.0 ng/ml in survivors) (53), indicating that the disease lethality may be due to virally driven hyperinflammation.

A report on SARS-CoV-2 showed that more than 70% of affected patients needed mechanical ventilation, and about 67% suffered from ARDS. Additionally, the number of death of the elderly patients with ARDS was considerably higher (57). As we mentioned above, the main variation in ARDS is the pulmonary and interstitial tissue injury due to non-specific cell infiltration, and the pivotal factor is the local excessive cytokine release, which has lead to pathological alteration in the whole body and clinical symptoms (37, 58).

The cytokine storm is thus at the basis of the onset and progression of ARDS. The serum levels of cytokines are considerably elevated in these patients, and the degree is clearly associated with death rate (16). The cytokine storm is also at the basis of the clinical progression of extrapulmonary multi-organ collapse (37, 59). This partly explicates the extra-pulmonary organ failure (i.e., elevated liver enzymes and creatinine) found in a few COVID-19 patients that do not show respiratory problems, indicating that the cytokine storm is the trigger of extrapulmonary injuries in tissues and organs.

**FIGURE 1:**
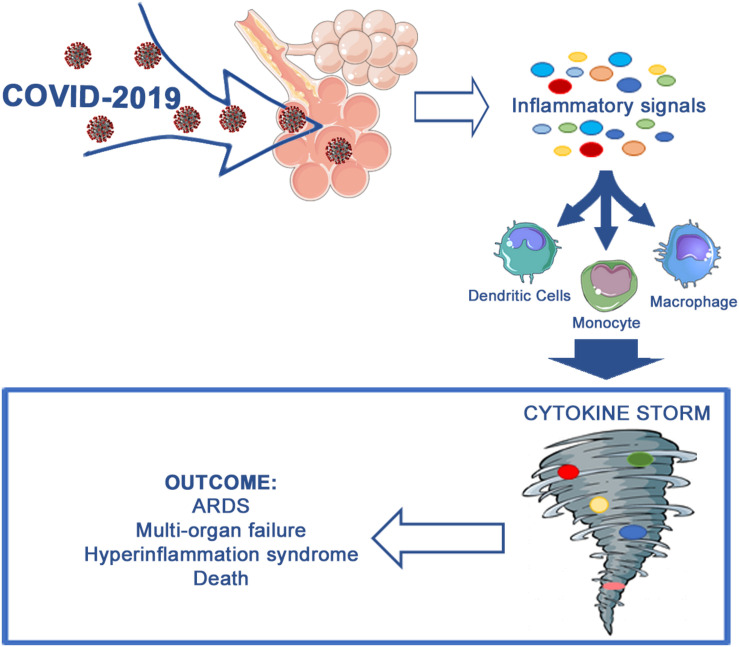
Aberrant immune host response occurring during COVID-19 infection.

In summary, the new coronavirus infection leads to an inflammatory cytokine storm in the affected patients. The cytokine storm, in turn, triggers ARDS and multi-organ failure and represents a crucial factor in COVID-19 exacerbation or even mortality (Figure 1).

However, the main question is why some patients are more predisposed to cytokine storm respect others. Different genetic mutations may also represent a risk factor for the severe disease course and the occurrence of cytokine storm in COVID-19. Notably, data obtained from a global population indicate that allelic alterations in cytokine genes showed a sharp latitudinal impact (60, 61). Geographical latitude is the main environmental factor that is affected by our evolutionary history with respect to environmental selection. The latitude is therefore associated with a variety of factors comprising genetic background, biometeorological factors, and socio−economic influences. Regarding the role of biometeorological factors, the sunlight has a pivotal role for the synthesis of Vitamin D, which in turn plays a key role in preserving the immune homeostasis. Genetic factors are known to account for up to 28% of inter−individual variability in serum 25(OH)D concentrations (62). Genetic as well as individual differences of vitamin D status have been reported across various populations (63). In the light of this, we can postulate that there is a possibility that vitamin D status may have some influence on geographical variance of COVID−19.

Furthermore, deficiency in vitamin D may lead to increased autoimmunity and elevated susceptibility to infections. Indeed, Vitamin D inhibit the production of proinflammatory cytokines (i.e., TNF−α and IFN−γ) and stimulate the release of anti−inflammatory cytokines. Vitamin D decreases the risk of microbial infection and death through different mechanism. A recent review categorized those mechanisms into three groups, including a physical barrier as well as innate and adaptative immunity (64). COVID-19 viruses disrupt junction integrity, increasing the susceptibility to the infection by the virus and other microorganisms (65), while vitamin D supports the maintenance of cell junctions integrity (66). Vitamin D may be valuable in controlling the cytokine storm and the outcome of COVID−2019 patients. Its deficiency leads to greater risk, and supplements of Vitamin D could thus be potentially used (67).

Cytokine regulation, however, depends on different upstream regulators, such as Toll-like Receptors (TLRs), and these interrelate with other components of innate immune system, such as complement elements. TLRs are a family of innate immune sensor proteins exerting a key function in infection, inflammation and immunity processes (68); TLR pathway may be significantly implicated in cytokine storm occurring during COVID-19 infection. To date, there are no studies regarding the role of TLR signaling in SARS−CoV−2 infection. Previous studies indicate, however, that genetic variation within TLRs or TLR signaling affected SARS−CoV infection (62, 68–71).

Moreover, the complement system interacts with TLRs, and it is thus involved in higher susceptibility to the infection and cytokine storm activation (72). In fact, a recent study reported that the complement system represents a crucial host mediator of SARS−CoV infection. SARS−CoV−infected *C3^–/–^* mice exhibited less respiratory impairment and lowered levels of chemokines and cytokines in the organs (73). In addition, hyperactivation of the complement system was reported in COVID−19 patients, and the highly pathogenic coronavirus N protein exacerbated MASP−2−mediated complement activation (74). Overall, the complement system is crucially involved in the stimulation of the cytokine storm and inflammation in SARS−CoV−2 infection.

## COVID-19 Experimental and Clinical Investigations

Data concerning the correlation between COVID-19 and cytokine/chemokine dysregulation are still limited, but the current available *in vitro* and clinical studies suggest a likeness with what was reported after SARS and MERS infections.

So far, few studies into SARS-CoV-2 infection have been reported. One interesting study compared SARS-CoV-2 and SARS-CoV behavior in the pulmonary tissue. The research group inoculated the viruses in *ex vivo* human pulmonary tissue samples and reported that SARS-CoV-2 was more efficient than SARS-CoV in both replicating and infecting human lung tissues. Additionally, SARS-CoV-2 infection was less competent in inducing the expression of any IFNs, suggesting that SARS-CoV and SARS-CoV-2 may differ in their capability to control proinflammatory cytokines and chemokines release. Indeed, SARS-CoV infection increased 11 out of the 13 proinflammatory factors tested in this study, while SARS-CoV-2 upregulated only five of them (i.e., CXCL10, IL6, CCL2, CXCL1, and CXCL5) despite replicating more efficiently. The expression of 12 out of 19 among IFNs and cytokines/chemokines genes tested was substantially lower in SARS-CoV-2-infected human samples than SARS-CoV-infected samples. Notably, CXCL8 transcription was increased only by SARS-CoV, but not SARS-CoV-2 infection, while the opposite for CXCL10 was detected (75).

Another research group isolated SARS-CoV-2 from a patient with established COVID-19 and compared virus tropism and replication competence with SARS, MERS, and 2009 pandemic influenza H1N1 (H1N1pdm) in *ex vivo* samples of human lung and bronchus. To assess extrapulmonary infection, the authors used *ex vivo* cultures of human conjunctiva epithelium (potential portals of infection for SARS-CoV-2) and human colorectal adenocarcinoma cell lines (17). SARS-CoV-2 was able to infect mucus-secreting, ciliated, and club cells of bronchial epithelium type 1 pneumocytes in the lung and the conjunctival mucosa. In the bronchus, SARS-CoV-2 replication was higher than SARS and similar to MERS and lower than H1N1pdm. In the lungs, SARS-CoV-2 replication was comparable to SARS and H1N1pdm but lower than MERS. In conjunctiva, SARS-CoV-2 replication was superior to SARS-CoV. SARS-CoV-2 was less effective in inducing proinflammatory cytokines than H1N1 and MERS. Both SARS-CoV and SARS-CoV-2 are thus comparably replicated in the alveolar epithelium; SARS-CoV-2 is replicated more extensively in the bronchus than SARS-CoV. These findings support valuable insights into the transmissibility of SARS-CoV-2 infection and dissimilarities with other respiratory pathogens (76).

In a retrospective study, the clinical and immunological features of 21 patients (17 male and four female) affected by COVID-19 were evaluated. These patients were classified in different degrees of severity, according to the guidelines of the National Health Commission of China. In particular, the 11 patients with severe form exhibited considerably elevated serum levels of IL-6, IL-10, and TNF-α in parallel to the reduced absolute number of T lymphocytes, CD4 + T cells, and CD8 + T cells with respect with moderate cases. This retrospective observational study suggests that SARS-CoV-2 infection may involve principally T lymphocytes, particularly CD4 + and CD8 + T cells, leading to decreased T lymphocytes number as well as IFN-γ production by CD4 + T cells. These potential immunological markers can be relevant due to their association with COVID-19 disease severity (6).

To characterize the transcriptional signatures of host inflammatory response to SARS-CoV-2, Xiong and collaborators performed a transcriptome sequencing of different proinflammatory genes from RNAs isolated from the broncho-alveolar lavage fluid and peripheral blood mononuclear cells of COVID-19 patients. This analysis showed distinct host inflammatory cytokine profiles to SARS-CoV-2 infection and supports the association between COVID-19 pathogenesis and aberrant cytokine release; CXCL10 in particular was upregulated in peripheral blood mononuclear cells, but no up-regulation of CXCL10 gene in broncho-alveolar lavage fluid was detected. Additionally, SARS-CoV-2 induced the activation in lymphocytes of numerous genes involved in apoptosis and P53 pathways, leading to the assumption that this activity may be the primary cause of lymphopenia frequently detected in COVID-19 cases. The transcriptome sequencing analysis of COVID-19 patients represents a significant source for clinical guidance on anti-inflammatory treatment and to understand the molecular mechanisms of host response (77).

Another study, involving 65 SARS-CoV-2-positive patients, revealed that the absolute numbers of CD4 + and CD8 + T cells and B cells progressively diminished in relation to increased severity of disease (78). Furthermore, Yang and collaborators analyzed 48 circulating cytokines from 53 COVID-19 patients (34 severe cases), and 14 resulted higher in patients with severe COVID-19 clinical history. Among them, CXCL10, CCL7, and IL-1 receptor antagonist were the ones strongly related to severity illness and, even more significantly, CXCL10 levels were the only one to be positively and significantly correlated with the viral load (79).

In 70 patients who survived severe COVID-19 pneumonia, 66 showed significant damage as revealed by CT scans taken before hospital release. The injury varied from dense clumps of tissue obstructing blood vessels of the alveoli to tissue lesions. The tissue lesions may represent signs of chronic lung disease and may be irreversible, rendering the patient frail (80). Furthermore, people who survived ARDS due to COVID-19 may have lasting pulmonary scarring (81). If pulmonary tissues are replaced with scar tissues, they are no longer functional as normal lung tissues, which may lead to poor gas exchange. Similar damage has been documented also in survivors of MERS and SARS even if those illnesses attacked only one lung.

Many patients hospitalized for COVID-19 also face cardiovascular problems, with unexpectedly high rates of blood clots, due to inflammatory reactions to this infection that lead to stroke, heart attack, lung blockages, neurological problems, and other complications with serious and lasting effects (82–86).

## Potential Treatments

The use of glucocorticoids represents one of the approaches to treat COVID-19 patients (87). The dosage and timing of administration are crucial to the outcome, especially of severe cases. Indeed, a too early administration of glucocorticoids impedes the immune system activation, thus enhancing the viral cargo and increasing the adverse effects. Consequently, glucocorticoids are mostly utilized in critical COVID-19 patients experiencing an inflammatory cytokine storm.

The inhibition of the aberrant inflammation through timely administration of glucocorticoids in the early stage of an inflammatory cytokine storm may efficiently inhibit ARDS onset and preserve the organs functions. For cases with progressive worsening of oxygenation indicators, rapid imaging progress, and aberrant inflammatory response, the use of glucocorticoid in the short term (3–5 days) is suitable, and the recommended dose is no more than equivalent to methylprednisolone 1–2 mg/kg/day (87). On the contrary, glucocorticoid at high dosage may impede the clearance of COVID-19 due to immunosuppression.

Notably, cytokines inhibition approaches are presently being investigated for COVID-19 treatment, and hydroxychloroquine, a long-known drug used as treatment of immune-mediated inflammatory diseases, showed high efficacy, reducing the time to clinical recovery and helping the absorption of pneumonia, as reported in a randomized clinical trial (88). Furthermore, it was able to prevent the release of TNF and IL-6 (89). Chloroquine phosphate has been used in the treatment of adults aged 18–65 in China (90). Based on ongoing analysis and emerging scientific data, however, the Food and Drug Administration (FDA) has revoked the emergency use authorization (EUA) to use chloroquine and hydroxychloroquine to treat COVID-19 in specific hospitalized patients under careful heart monitoring. FDA made this decision based on recent findings from a large, randomized clinical trial in hospitalized and non-hospitalized patients that revealed that these drugs had no benefit for improving the recovery and decreasing the death (91, 92).

During the cytokine storm, the most relevant cytokines are the IL-1 family; studies that focus on the inhibition of IL-1β to counteract the cytokine storm attracted most attention. Interestingly, Anakinra, an antagonist of IL-1β, radically ameliorated the survival rate of patients with severe sepsis (93). However, there are no clinical studies to treat COVID-19 using specific IL-1 family blockers, and *in vivo* studies and clinical trials are thus necessary.

Regarding the other ILs, Tocilizumab, a humanized anti-IL-6 receptor IgG monoclonal antibody applied as treatment for chronic inflammatory diseases, was used as treatment option to better understand the underlying molecular mechanism of aberrant cytokine response in COVID-19 pneumonia and to define the clinical effects. In a very recent study, upon use of Tocilizumab, 83% of cases showed remarkable clinical and laboratory ameliorations, while 17% of the patients needed short-term ventilator assistance in the intensive care unit. This study suggested that Tocilizumab (administered at the right time) is valuable in inhibiting the injury caused by aberrant cytokine response and offers clinical and radiological recovery. Indeed, upon use of Tocilizumab, all patients showed normalized arterial oxygen saturation levels and the eosinophil values increased significantly in response to the treatment (94). Furthermore, Tocilizumab impedes the IL-6-mediated signal transduction by blocking the IL-6 receptor interaction. Clinical data on the use of Tocilizumab in COVID-19 cases are still limited; however, some authors propose its use in SARS-CoV-2 patients with elevated IL-6 levels (36, 37).

Another drug tested for COVID-19 was Sarilumab, an IL-6R antibody. There are contrasting data regarding its therapeutic potential. For instance, an observational study reported that IL-6R inhibitors, administered prior 45% FiO_2_ (fraction of inspired oxygen) requirement, improved Covid-19 outcomes (95). On the other hand, recently, leading companies announced that the US Phase 3 trial of Sarilumab (400 mg) in COVID-19 patients requiring mechanical ventilation did not meet its primary and key secondary endpoints. In particular, minor positive trends that did not reach statistical significance were observed in the primary pre-specified analysis group (critical patients mechanically ventilated at baseline), and these were opposed by negative trends in a subgroup of critical patients who were not mechanically ventilated at baseline. Serious adverse effects that occurred in at least 3% of patients upon Sarilumab treatment were multi-organ failure syndrome and hypotension. Based on these results, the trial has been stopped (96).

## Conclusion

Aberrant immune host response together with cytokine storm and lymphocytopenia, followed by ARDS, are still relevant problems that affect the severity of COVID-19, and the modulation of the immune response and inflammation may thus be considered as crucial. Although the above-mentioned therapeutic approaches presented encouraging results, further studies are necessary in order to better understand the immune response and immunopathogenesis occurring during COVID-19 infection. Moreover, in the light of the reported studies, many people that have contracted the COVID-19, also after recovery, need to be considered as frail patients, especially the ones in which ARSD and consequent multi-organ failure occurred.

## Author Contributions

VC wrote the manuscript upon CF supervision. VC prepared the figure. AC substantially revised the manuscript. All authors contributed to the article and approved the submitted version.

## Conflict of Interest

The authors declare that the research was conducted in the absence of any commercial or financial relationships that could be construed as a potential conflict of interest.

## References

[1] Perlman S. Another decade, another Coronavirus. *N Engl J Med.* (2020) 382:760–2. 10.1056/NEJMe2001126 31978944 PMC7121143

[2] Astuti I Ysrafill Severe acute respiratory syndrome Coronavirus 2 (SARS-CoV-2): an overview of viral structure and host response. *Diabetes Metab Syndr.* (2020) 14:407–12. 10.1016/j.dsx.2020.04.020 32335367 PMC7165108

[3] Zhu N Zhang D Wang W Li X Yang B Song J A novel Coronavirus from patients with pneumonia in China, 2019. *N Engl J Med.* (2020) 382:727–33. 10.1056/NEJMoa2001017 31978945 PMC7092803

[4] WHO *WHO Coronavirus Disease (COVID-19) Dashboard.* Available online at: https://covid19.who.int/ (accessed July 31, 2020).

[5] Richardson S Hirsch JS Narasimhan M Crawford JM McGinn T Davidson KW Presenting characteristics, comorbidities, and outcomes among 5700 patients hospitalized with COVID-19 in the New York City area. *JAMA.* (2020) 323:2052–9. 10.1001/jama.2020.6775 32320003 PMC7177629

[6] Chen G Wu D Guo W Cao Y Huang D Wang H Clinical and immunological features of severe and moderate Coronavirus disease 2019. *J Clin Investig.* (2020) 130:2620–9. 10.1172/JCI137244 32217835 PMC7190990

[7] Onder G Rezza G Brusaferro S. Case-fatality rate and characteristics of patients dying in relation to COVID-19 in Italy. *JAMA.* (2020) 323:1775–1776. 10.1001/jama.2020.4683 32203977

[8] Chen T Wu D Chen H Yan W Yang D Chen G Clinical characteristics of 113 deceased patients with coronavirus disease 2019: retrospective study. *BMJ.* (2020) 368:m1091. 10.1136/bmj.m1091 32217556 PMC7190011

[9] Chen N Zhou M Dong X Qu J Gong F Han Y Epidemiological and clinical characteristics of 99 cases of 2019 novel coronavirus pneumonia in Wuhan, China: a descriptive study. *Lancet.* (2020) 395:507–13. 10.1016/S0140-6736(20)30211-7 32007143 PMC7135076

[10] Gong J Dong H Xia SQ Huang YZ Wang D Zhao Y Correlation analysis between disease severity and inflammation-related parameters in patients with COVID-19 pneumonia. *Infect Dis (except HIV/AIDS).* (2020). 10.1101/2020.02.25.20025643 [Epub ahead of print]. PMC7750784 33349241

[11] Cron RQ Chatham WW. The rheumatologist’s role in COVID-19. *J Rheumatol.* (2020) 47:639–42. 10.3899/jrheum.200334 32209661

[12] Perlman S Netland J. Coronaviruses post-SARS: update on replication and pathogenesis. *Nat Rev Microbiol.* (2009) 7:439–50. 10.1038/nrmicro2147 19430490 PMC2830095

[13] Heugel J Martin ET Kuypers J Englund JA. Coronavirus-associated pneumonia in previously healthy children. *Pediatr Infect Dis J.* (2007) 26:753–5. 10.1097/INF.0b013e318054e31b 17848893

[14] Kuypers J Martin ET Heugel J Wright N Morrow R Englund JA. Clinical disease in children associated with newly described coronavirus subtypes. *Pediatrics.* (2007) 119:e70–6. 10.1542/peds.2006-1406 17130280

[15] Hadjadj J Yatim N Barnabei L Corneau A Boussier J Pere H Impaired type I interferon activity and exacerbated inflammatory responses in severe Covid-19 patients. *Infect Dis (except HIV/AIDS).* (2020) 369:718–24. 10.1101/2020.04.19.20068015 PMC7402632 32661059

[16] Mehta P McAuley DF Brown M Sanchez E Tattersall RS Manson JJ. COVID-19: consider cytokine storm syndromes and immunosuppression. *Lancet.* (2020) 395:1033–4. 10.1016/S0140-6736(20)30628-0 32192578 PMC7270045

[17] Xu Z Shi L Wang Y Zhang J Huang L Zhang C Pathological findings of COVID-19 associated with acute respiratory distress syndrome. *Lancet Respir Med.* (2020) 8:420–2. 10.1016/S2213-2600(20)30076-X 32085846 PMC7164771

[18] Channappanavar R Fehr AR Vijay R Mack M Zhao J Meyerholz DK Dysregulated type I interferon and inflammatory monocyte-macrophage responses cause lethal pneumonia in SARS-CoV-infected mice. *Cell Host Microbe.* (2016) 19:181–93. 10.1016/j.chom.2016.01.007 26867177 PMC4752723

[19] Davidson S Maini MK Wack A. Disease-promoting effects of type I interferons in viral, bacterial, and coinfections. *J Interferon Cytokine Res.* (2015) 35:252–64. 10.1089/jir.2014.0227 25714109 PMC4389918

[20] Shaw AC Goldstein DR Montgomery RR. Age-dependent dysregulation of innate immunity. *Nat Rev Immunol.* (2013) 13:875–87. 10.1038/nri3547 24157572 PMC4096436

[21] Chakraborty RK Burns B. *Systemic Inflammatory Response Syndrome.* Treasure Island, FL: StatPearls Publishing (2020). 31613449

[22] Yazdanpanah F Hamblin MR Rezaei N. The immune system and COVID-19: Friend or foe? *Life Sci.* (2020) 256:117900. 10.1016/j.lfs.2020.117900 32502542 PMC7266583

[23] Law HKW Cheung CY Ng HY Sia SF Chan YO Luk W Chemokine up-regulation in SARS-coronavirus-infected, monocyte-derived human dendritic cells. *Blood.* (2005) 106:2366–74. 10.1182/blood-2004-10-4166 15860669 PMC1895271

[24] Lau SKP Lau CCY Chan K-H Li CPY Chen H Jin D-Y Delayed induction of proinflammatory cytokines and suppression of innate antiviral response by the novel Middle East respiratory syndrome coronavirus: implications for pathogenesis and treatment. *J Gen Virol.* (2013) 94:2679–90. 10.1099/vir.0.055533-0 24077366

[25] Tynell J Westenius V Rönkkö E Munster VJ Melén K Österlund P Middle East respiratory syndrome coronavirus shows poor replication but significant induction of antiviral responses in human monocyte-derived macrophages and dendritic cells. *J Gen Virol.* (2016) 97:344–55. 10.1099/jgv.0.000351 26602089 PMC4804640

[26] Ye Q Wang B Mao J. The pathogenesis and treatment of the ‘Cytokine Storm’ in COVID-19. *J Infect.* (2020) 80:607–13. 10.1016/j.jinf.2020.03.037 32283152 PMC7194613

[27] Zhou J Chu H Li C Wong BH-Y Cheng Z-S Poon VK-M Active replication of Middle East respiratory syndrome coronavirus and aberrant induction of inflammatory cytokines and chemokines in human macrophages: implications for pathogenesis. *J Infect Dis.* (2014) 209:1331–42. 10.1093/infdis/jit504 24065148 PMC7107356

[28] Scheuplein VA Seifried J Malczyk AH Miller L Höcker L Vergara-Alert J High secretion of interferons by human plasmacytoid dendritic cells upon recognition of Middle East respiratory syndrome coronavirus. *J Virol.* (2015) 89:3859–69. 10.1128/JVI.03607-14 25609809 PMC4403407

[29] Min C-K Cheon S Ha N-Y Sohn KM Kim Y Aigerim A Comparative and kinetic analysis of viral shedding and immunological responses in MERS patients representing a broad spectrum of disease severity. *Sci Rep.* (2016) 6:25359. 10.1038/srep25359 27146253 PMC4857172

[30] Ng DL Al Hosani F Keating MK Gerber SI Jones TL Metcalfe MG Clinicopathologic, immunohistochemical, and ultrastructural findings of a fatal case of middle east respiratory syndrome Coronavirus infection in the United Arab Emirates, April 2014. *Am J Pathol.* (2016) 186:652–8. 10.1016/j.ajpath.2015.10.024 26857507 PMC7093852

[31] Chien J-Y Hsueh P-R Cheng W-C Yu C-J Yang P-C. Temporal changes in cytokine/chemokine profiles and pulmonary involvement in severe acute respiratory syndrome. *Respirology.* (2006) 11:715–22. 10.1111/j.1440-1843.2006.00942.x 17052299 PMC7192207

[32] Wang C-H Liu C-Y Wan Y-L Chou C-L Huang K-H Lin H-C Persistence of lung inflammation and lung cytokines with high-resolution CT abnormalities during recovery from SARS. *Respir Res.* (2005) 6:42. 10.1186/1465-9921-6-42 15888207 PMC1156954

[33] Wong CK Lam CWK Wu AKL Ip WK Lee NLS Chan IHS Plasma inflammatory cytokines and chemokines in severe acute respiratory syndrome. *Clin Exp Immunol.* (2004) 136:95–103. 10.1111/j.1365-2249.2004.02415.x 15030519 PMC1808997

[34] Smits SL de Lang A van den Brand JMA Leijten LM van IJcken WF Eijkemans MJC Exacerbated innate host response to SARS-CoV in aged non-human primates. *PLoS Pathog.* (2010) 6:e1000756. 10.1371/journal.ppat.1000756 20140198 PMC2816697

[35] Rockx B Baas T Zornetzer GA Haagmans B Sheahan T Frieman M Early upregulation of acute respiratory distress syndrome-associated cytokines promotes lethal disease in an aged-mouse model of severe acute respiratory syndrome coronavirus infection. *J Virol.* (2009) 83:7062–74. 10.1128/JVI.00127-09 19420084 PMC2704758

[36] Zhang C Wu Z Li J-W Zhao H Wang G-Q. Cytokine release syndrome in severe COVID-19: interleukin-6 receptor antagonist tocilizumab may be the key to reduce mortality. *Int J Antimicrob Agents.* (2020) 55:105954. 10.1016/j.ijantimicag.2020.105954 32234467 PMC7118634

[37] Coperchini F Chiovato L Croce L Magri F Rotondi M. The cytokine storm in COVID-19: an overview of the involvement of the chemokine/chemokine-receptor system. *Cytokine Growth Factor Rev.* (2020) 53:25–32. 10.1016/j.cytogfr.2020.05.003 32446778 PMC7211650

[38] Huang C Wang Y Li X Ren L Zhao J Hu Y Clinical features of patients infected with 2019 novel coronavirus in Wuhan, China. *Lancet.* (2020) 395:497–506. 10.1016/S0140-6736(20)30183-5 31986264 PMC7159299

[39] Marchingo JM Sinclair LV Howden AJ Cantrell DA. Quantitative analysis of how Myc controls T cell proteomes and metabolic pathways during T cell activation. *Elife.* (2020) 9:e53725. 10.7554/eLife.53725 32022686 PMC7056270

[40] Diao B Wang C Tan Y Chen X Liu Y Ning L Reduction and functional exhaustion of T cells in patients with Coronavirus disease 2019 (COVID-19). *Front Immunol.* (2020) 11:827. 10.3389/fimmu.2020.00827 32425950 PMC7205903

[41] Zheng M Gao Y Wang G Song G Liu S Sun D Functional exhaustion of antiviral lymphocytes in COVID-19 patients. *Cell Mol Immunol.* (2020) 17:533–5. 10.1038/s41423-020-0402-2 32203188 PMC7091858

[42] Hossein-Khannazer N Shokoohian B Shpichka A Aghdaei HA Timashev P Vosough M. Novel therapeutic approaches for treatment of COVID-19. *J Mol Med.* (2020) 98:789–803. 10.1007/s00109-020-01927-6 32494931 PMC7268974

[43] Herold S Steinmueller M von Wulffen W Cakarova L Pinto R Pleschka S Lung epithelial apoptosis in influenza virus pneumonia: the role of macrophage-expressed TNF-related apoptosis-inducing ligand. *J Exp Med.* (2008) 205:3065–77. 10.1084/jem.20080201 19064696 PMC2605231

[44] Högner K Wolff T Pleschka S Plog S Gruber AD Kalinke U Macrophage-expressed IFN-β contributes to apoptotic alveolar epithelial cell injury in severe influenza virus pneumonia. *PLoS Pathog.* (2013) 9:e1003188. 10.1371/journal.ppat.1003188 23468627 PMC3585175

[45] Rodrigue-Gervais IG Labbé K Dagenais M Dupaul-Chicoine J Champagne C Morizot A Cellular inhibitor of apoptosis protein cIAP2 protects against pulmonary tissue necrosis during influenza virus infection to promote host survival. *Cell Host Microbe.* (2014) 15:23–35. 10.1016/j.chom.2013.12.003 24439895

[46] Drosten C Seilmaier M Corman VM Hartmann W Scheible G Sack S Clinical features and virological analysis of a case of Middle East respiratory syndrome coronavirus infection. *Lancet Infect Dis.* (2013) 13:745–51. 10.1016/S1473-3099(13)70154-3 23782859 PMC7164791

[47] Lew TWK Kwek T-K Tai D Earnest A Loo S Singh K Acute respiratory distress syndrome in critically ill patients with severe acute respiratory syndrome. *JAMA.* (2003) 290:374–80. 10.1001/jama.290.3.374 12865379

[48] Cameron MJ Bermejo-Martin JF Danesh A Muller MP Kelvin DJ. Human immunopathogenesis of severe acute respiratory syndrome (SARS). *Virus Res.* (2008) 133:13–9. 10.1016/j.virusres.2007.02.014 17374415 PMC7114310

[49] Jiang Y Xu J Zhou C Wu Z Zhong S Liu J Characterization of cytokine/chemokine profiles of severe acute respiratory syndrome. *Am J Respir Crit Care Med.* (2005) 171:850–7. 10.1164/rccm.200407-857OC 15657466

[50] Reghunathan R Jayapal M Hsu L-Y Chng H-H Tai D Leung BP Expression profile of immune response genes in patients with severe acute respiratory syndrome. *BMC Immunol.* (2005) 6:2. 10.1186/1471-2172-6-2 15655079 PMC546205

[51] Zhonghua L Xing B Xue Z. [An update on the epidemiological characteristics of novel coronavirus pneumonia (COVID-19)]. Epidemiology Working Group for NCIP Epidemic Response, Chinese Center for Disease Control and Prevention (2020) 41:139–44. 10.3760/cma.j.issn.0254-6450.2020.02.002 32057211

[52] Chousterman BG Swirski FK Weber GF. Cytokine storm and sepsis disease pathogenesis. *Semin Immunopathol.* (2017) 39:517–28. 10.1007/s00281-017-0639-8 28555385

[53] Ruan Q Yang K Wang W Jiang L Song J. Clinical predictors of mortality due to COVID-19 based on an analysis of data of 150 patients from Wuhan, China. *Intensive Care Med.* (2020) 46:846–8. 10.1007/s00134-020-05991-x 32125452 PMC7080116

[54] Ramos-Casals M Brito-Zerón P López-Guillermo A Khamashta MA Bosch X. Adult haemophagocytic syndrome. *Lancet.* (2014) 383:1503–16. 10.1016/S0140-6736(13)61048-X 24290661

[55] Karakike E Giamarellos-Bourboulis EJ. Macrophage activation-like syndrome: a distinct entity leading to early death in sepsis. *Front Immunol.* (2019) 10:55. 10.3389/fimmu.2019.00055 30766533 PMC6365431

[56] Seguin A Galicier L Boutboul D Lemiale V Azoulay E. Pulmonary involvement in patients with hemophagocytic lymphohistiocytosis. *Chest.* (2016) 149:1294–301. 10.1016/j.chest.2015.11.004 26836913

[57] Yang X Yu Y Xu J Shu H Xia J Liu H Clinical course and outcomes of critically ill patients with SARS-CoV-2 pneumonia in Wuhan, China: a single-centered, retrospective, observational study. *Lancet Respir Med.* (2020) 8:475–81. 10.1016/S2213-2600(20)30079-5 32105632 PMC7102538

[58] Douda DN Jackson R Grasemann H Palaniyar N. Innate immune collectin surfactant protein D simultaneously binds both neutrophil extracellular traps and carbohydrate ligands and promotes bacterial trapping. *J Immunol.* (2011) 187:1856–65. 10.4049/jimmunol.1004201 21724991

[59] Wang H Ma S. The cytokine storm and factors determining the sequence and severity of organ dysfunction in multiple organ dysfunction syndrome. *Am J Emerg Med.* (2008) 26:711–5. 10.1016/j.ajem.2007.10.031 18606328

[60] Srinivas L Vellichirammal NN Alex AM Nair C Nair IV Banerjee M. Pro-inflammatory cytokines and their epistatic interactions in genetic susceptibility to schizophrenia. *J Neuroinflammation.* (2016) 13:105. 10.1186/s12974-016-0569-8 27177030 PMC4866417

[61] Debnath M Banerjee M Berk M. Genetic gateways to COVID−19 infection: implications for risk, severity, and outcomes. *FASEB J.* (2020) 34:8787–95. 10.1096/fj.202001115R 32525600 PMC7300732

[62] Shea MK Benjamin EJ Dupuis J Massaro JM Jacques PF D’Agostino RB Genetic and non-genetic correlates of vitamins K and D. *Eur J Clin Nutr.* (2009) 63:458–64. 10.1038/sj.ejcn.1602959 18030310 PMC2681093

[63] Lips P Cashman KD Lamberg-Allardt C Bischoff-Ferrari HA Obermayer-Pietsch B Bianchi ML Current vitamin D status in European and Middle East countries and strategies to prevent vitamin D deficiency: a position statement of the European calcified tissue society. *Eur J Endocrinol.* (2019) 180:23–54. 10.1530/EJE-18-0736 30721133

[64] Rondanelli M Miccono A Lamburghini S Avanzato I Riva A Allegrini P Self-care for common colds: the pivotal role of vitamin D, vitamin C, zinc, and *Echinacea* in three main immune interactive clusters (Physical Barriers, Innate and Adaptive Immunity) involved during an episode of common colds—practical advice on dosages and on the time to take these Nutrients/Botanicals in order to prevent or treat common colds. *Evid Based Complement Alternat Med.* (2018) 2018:1–36. 10.1155/2018/5813095 29853961 PMC5949172

[65] Rossi GA Fanous H Colin AA. Viral strategies predisposing to respiratory bacterial superinfections. *Pediatr Pulmonol.* (2020) 55:1061–73. 10.1002/ppul.24699 32084305

[66] Schwalfenberg GK. A review of the critical role of vitamin D in the functioning of the immune system and the clinical implications of vitamin D deficiency. *Mol Nutr Food Res.* (2011) 55:96–108. 10.1002/mnfr.201000174 20824663

[67] Grant WB Lahore H McDonnell SL Baggerly CA French CB Aliano JL Evidence that vitamin D supplementation could reduce risk of influenza and COVID-19 infections and deaths. *Nutrients.* (2020) 12:988. 10.3390/nu12040988 32252338 PMC7231123

[68] Sallenave J-M Guillot L. Innate immune signaling and proteolytic pathways in the resolution or exacerbation of SARS-CoV-2 in Covid-19: key therapeutic targets? *Front Immunol.* (2020) 11:1229. 10.3389/fimmu.2020.01229 32574272 PMC7270404

[69] Totura AL Whitmore A Agnihothram S Schäfer A Katze MG Heise MT Toll-like receptor 3 signaling via TRIF contributes to a protective innate immune response to severe acute respiratory syndrome Coronavirus infection. *mBio.* (2015) 6:e00638-15. 10.1128/mBio.00638-15 26015500 PMC4447251

[70] Gralinski LE Menachery VD Morgan AP Totura AL Beall A Kocher J Allelic variation in the toll-like receptor adaptor protein *Ticam2* contributes to SARS-Coronavirus pathogenesis in mice. *G3.* (2017) 7:1653–63. 10.1534/g3.117.041434 28592648 PMC5473747

[71] Lim HK Huang SXL Chen J Kerner G Gilliaux O Bastard P Severe influenza pneumonitis in children with inherited TLR3 deficiency. *J Exp Med.* (2019) 216:2038–56. 10.1084/jem.20181621 31217193 PMC6719423

[72] Stoermer KA Morrison TE. Complement and viral pathogenesis. *Virology.* (2011) 411:362–73. 10.1016/j.virol.2010.12.045 21292294 PMC3073741

[73] Gralinski LE Sheahan TP Morrison TE Menachery VD Jensen K Leist SR Complement activation contributes to severe acute respiratory syndrome Coronavirus pathogenesis. *mBio.* (2018) 9:e01753-18. 10.1128/mBio.01753-18 30301856 PMC6178621

[74] Gao T Hu M Zhang X Li H Zhu L Liu H Highly pathogenic coronavirus N protein aggravates lung injury by MASP-2-mediated complement over-activation. *Infect Dis (except HIV/AIDS).* (2020). 10.1101/2020.03.29.20041962 [Epub ahead of print].

[75] Chu H Chan JF-W Wang Y Yuen TT-T Chai Y Hou Y Comparative replication and immune activation profiles of SARS-CoV-2 and SARS-CoV in human lungs: an ex vivo study with implications for the pathogenesis of COVID-19. *Clin Infect Dis.* (2020):ciaa410. 10.1093/cid/ciaa410 [Epub ahead of print]. 32270184 PMC7184390

[76] Hui KPY Cheung M-C Perera RAPM Ng K-C Bui CHT Ho JCW Tropism, replication competence, and innate immune responses of the coronavirus SARS-CoV-2 in human respiratory tract and conjunctiva: an analysis in ex-vivo and in-vitro cultures. *Lancet Respir Med.* (2020) 8:687–95. 10.1016/S2213-2600(20)30193-4 32386571 PMC7252187

[77] Xiong Y Liu Y Cao L Wang D Guo M Jiang A Transcriptomic characteristics of bronchoalveolar lavage fluid and peripheral blood mononuclear cells in COVID-19 patients. *Emerg Microbes Infect.* (2020) 9:761–70. 10.1080/22221751.2020.1747363 32228226 PMC7170362

[78] Wang F Hou H Luo Y Tang G Wu S Huang M The laboratory tests and host immunity of COVID-19 patients with different severity of illness. *JCI Insight.* (2020) 5:e137799. 10.1172/jci.insight.137799 32324595 PMC7259533

[79] Yang Y Shen C Li J Yuan J Yang M Wang F Exuberant elevation of IP-10, MCP-3 and IL-1ra during SARS-CoV-2 infection is associated with disease severity and fatal outcome. *Infect Dis (except HIV/AIDS).* (2020). 10.1101/2020.03.02.20029975 [Epub ahead of print].

[80] Wang Y Dong C Hu Y Li C Ren Q Zhang X Temporal changes of CT findings in 90 patients with COVID-19 pneumonia: a longitudinal study. *Radiology.* (2020) 296:E55–64. 10.1148/radiol.2020200843 32191587 PMC7233482

[81] Spagnolo P Balestro E Aliberti S Cocconcelli E Biondini D Casa GD Pulmonary fibrosis secondary to COVID-19: a call to arms? *Lancet Respir Med.* (2020) 8:750–2. 10.1016/S2213-2600(20)30222-8 32422177 PMC7228737

[82] Frydman GH Boyer EW Nazarian RM Van Cott EM Piazza G. Coagulation status and venous thromboembolism risk in African Americans: a potential risk factor in COVID-19. *Clin Appl Thromb Hemost.* (2020) 26:1076029620943671. 10.1177/1076029620943671 32702995 PMC7383642

[83] Mangion K Morrow A Bagot C Bayes H Blyth KG Church C The chief scientist office cardiovascular and pulmonary imaging in SARS Coronavirus disease-19 (CISCO-19) study. *Cardiovasc Res.* (2020):cvaa209. 10.1093/cvr/cvaa209 [Epub ahead of print]. 32702087 PMC7454350

[84] Lingamaneni P Gonakoti S Moturi K Vohra I Zia M. Heparin-induced thrombocytopenia in COVID-19. *J Investig Med High Impact Case Rep.* (2020) 8:2324709620944091. 10.1177/2324709620944091 32720827 PMC7388103

[85] Mareev VY Orlova YA Pavlikova EP Matskeplishvili ST Krasnova TN Malahov PS [Steroid pulse -therapy in patients With coronAvirus Pneumonia (COVID-19), sYstemic inFlammation And Risk of vEnous thRombosis and thromboembolism (WAYFARER Study)]. *Kardiologiia.* (2020) 60:15–29. 10.18087/cardio.2020.6.n1226 32720612

[86] Gavriatopoulou M Korompoki E Fotiou D Ntanasis-Stathopoulos I Psaltopoulou T Kastritis E Organ-specific manifestations of COVID-19 infection. *Clin Exp Med.* (2020). 10.1007/s10238-020-00648-x [Epub ahead of print]. 32720223 PMC7383117

[87] Qin Y-Y Zhou Y-H Lu Y-Q Sun F Yang S Harypursat V Effectiveness of glucocorticoid therapy in patients with severe coronavirus disease 2019: protocol of a randomized controlled trial. *Chin Med J.* (2020) 133:1080–6. 10.1097/CM9.0000000000000791 32149773 PMC7147272

[88] Chen Z Hu J Zhang Z Jiang S Han S Yan D Efficacy of hydroxychloroquine in patients with COVID-19: results of a randomized clinical trial. *Epidemiology.* (2020). 10.1101/2020.03.22.20040758 [Epub ahead of print].

[89] Gao J Tian Z Yang X. Breakthrough: chloroquine phosphate has shown apparent efficacy in treatment of COVID-19 associated pneumonia in clinical studies. *Biosci Trends.* (2020) 14:72–3. 10.5582/bst.2020.01047 32074550

[90] Sun X Li S Li K Hu X. Pharmaceutical care of chloroquine phosphate in elderly patients with coronavirus pneumonia (COVID-19). *Aging Med.* (2020) 3:98–101. 10.1002/agm2.12104 32666027 PMC7338693

[91] Cavalcanti AB Zampieri FG Rosa RG Azevedo LCP Veiga VC Avezum A Hydroxychloroquine with or without Azithromycin in Mild-to-Moderate Covid-19. *N Engl J Med.* (2020):NEJMoa2019014. 10.1056/NEJMoa2019014 [Epub ahead of print]. 32706953 PMC7397242

[92] Skipper CP Pastick KA Engen NW Bangdiwala AS Abassi M Lofgren SM Hydroxychloroquine in nonhospitalized adults with early COVID-19: a randomized trial. *Ann Intern Med.* (2020):M20– 4207. 10.7326/M20-4207 [Epub ahead of print]. 32673060 PMC7384270

[93] Shakoory B Carcillo JA Chatham WW Amdur RL Zhao H Dinarello CA Interleukin-1 receptor blockade is associated with reduced mortality in sepsis patients with features of macrophage activation syndrome: reanalysis of a prior phase III trial. *Crit Care Med.* (2016) 44:275–81. 10.1097/CCM.0000000000001402 26584195 PMC5378312

[94] Borku Uysal B Ikitimur H Yavuzer S Ikitimur B Uysal H Islamoglu MS Tociluzumab challenge: a series of cytokine storm therapy experience in hospitalized Covid−19 pneumonia patients. *J Med Virol.* (2020):jmv.26111. 10.1002/jmv.26111 [Epub ahead of print]. 32484930 PMC7300754

[95] Sinha P Mostaghim A Bielick CG McLaughlin A Hamer DH Wetzler L Early administration of Interleukin-6 inhibitors for patients with severe Covid-19 disease is associated with decreased intubation, reduced mortality, and increased discharge. *Int J Infect Dis.* (2020). [Epub ahead of print]. 10.1016/j.ijid.2020.07.023 PMC7591937 32721528

[96] ClinicalTrials.gov *Evaluation of the Efficacy and Safety of Sarilumab in Hospitalized Patients With COVID-19.* (2020). Available online at: https://clinicaltrials.gov/ct2/show/NCT04315298 (accessed March 19, 2020).

